# Pellet-Based Fused Filament Fabrication (FFF)-Derived Process for the Development of Polylactic Acid/Hydroxyapatite Scaffolds Dedicated to Bone Regeneration

**DOI:** 10.3390/ma15165615

**Published:** 2022-08-16

**Authors:** Marie Bayart, Marie Dubus, Sébastien Charlon, Halima Kerdjoudj, Nicolas Baleine, Samira Benali, Jean-Marie Raquez, Jérémie Soulestin

**Affiliations:** 1 Centre for Materials and Processes, IMT Nord Europe, Institut Mines-Télécom, University of Lille, F-59000 Lille, France; 2 Biomatériaux et Inflammation en Site Osseux (BIOS) EA 4691 & UFR d’Odontologie, Université de Reims Champagne-Ardenne, F-51100 Reims, France; 3 Center of Innovation and Research in Materials and Polymers (CIRMAP), Laboratory of Polymeric and Composite Materials, University of Mons (UMons), Place du Parc 20, 7000 Mons, Belgium

**Keywords:** bone regeneration, scaffolds, fused filament fabrication (FFF), polylactic acid (PLA), hydroxyapatite

## Abstract

Scaffolds can be defined as 3D architectures with specific features (surface properties, porosity, rigidity, biodegradability, etc.) that help cells to attach, proliferate, and to differentiate into specific lineage. For bone regeneration, rather high mechanical properties are required. That is why polylactic acid (PLA) and PLA/hydroxyapatite (HA) scaffolds (10 wt.%) were produced by a peculiar fused filament fabrication (FFF)-derived process. The effect of the addition of HA particles in the scaffolds was investigated in terms of morphology, biological properties, and biodegradation behavior. It was found that the scaffolds were biocompatible and that cells managed to attach and proliferate. Biodegradability was assessed over a 5-month period (according to the ISO 13781-Biodegradability norm) through gel permeation chromatography (GPC), differential scanning calorimetry (DSC), and compression tests. The results revealed that the presence of HA in the scaffolds induced a faster and more complete polymer biodegradation, with a gradual decrease in the molar mass (Mn) and compressive mechanical properties over time. In contrast, the Mn of PLA only decreased during the processing steps to obtain scaffolds (extrusion + 3D-printing) but PLA scaffolds did not degrade during conditioning, which was highlighted by a high retention of the mechanical properties of the scaffolds after conditioning.

## 1. Introduction

Additive manufacturing (AM), also often named 3D-printing, can address many issues in the biomedical sector. AM gathers the different manufacturing processes that build 3D objects by adding materials layer by layer [1] and thus allows the fabrication of complex and personalized devices. Different AM techniques are used in the biomedical field, such as selective laser melting (SLM) and sintering (SLS), electron beam melting (EBM), and fused filament fabrication (FFF) that work by softening/melting the material, while stereolithography (SLA) consists of curing the raw material [2,3]. FFF is very popular as it is affordable, requires lower-cost materials than the other techniques, mostly thermoplastic polymers, and has high-dimensional accuracy and short cycle time [4].

Tissue engineering is an interdisciplinary and multidisciplinary research field that is growing exponentially over time, and scaffold materials and their fabrication techniques are key elements that are rapidly evolving [5]. Scaffolds for bone regenerative medicine and tissue engineering are 3D porous structures that should provide adequate space, structure, and degradability to allow for proper integration with the host tissue and, therefore, bone regeneration. While a macroporous structure is required for cell and tissue ingrowth, micropores generate a high surface area and are essential for better adsorption of bone-inducing proteins and ion exchange [6]. Osteoblasts (10–50 µm) are known to prefer large macropores [7] and sizes between 200 and 500 µm [8] and more generally >300 µm provide enough space for the colonization of osteoblasts, bone ingrowth, vascularization, and innervation [9]. In contrast to usual methods of scaffold production such as particulate/salt leaching, inducing random porosity, AM techniques allow for the development of scaffolds with easily tunable porosity and pore size. 

As bone matrix is a combination of organic (mainly collagen) and inorganic (apatite) composite materials, it seems logical to combine polymers and hydroxyapatite (HA) to develop scaffolds that meet all the desired requirements for bone applications [10,11,12,13,14,15]. HA is widely used in bone tissue engineering to enhance bioactivity, osteoconductivity, and osteoblast differentiation [16,17,18,19,20,21,22,23,24]. By combining HA with biodegradable polymers, it should be possible to achieve optimal results, with the aim of obtaining properties superior to those of the individual components. Biodegradability is necessary to limit the number of surgeries: once bone regeneration is complete, the implant is eliminated by the body without further invasive intervention.

Biodegradable thermoplastic polyesters such as polylactic acid (PLA), polyglycolic acid (PGA), their copolymer polylactic-co-glycolic acid (PLGA) and polycaprolactone (PCL) were used to produce scaffolds [25,26,27,28,29,30]. Among all these biodegradable polymers, PLA seems to be an appropriate candidate because of its dimensional stability that makes it ideal to be processed via AM [31]. Many PLA scaffolds, including FFF printed scaffolds and PLA/HA scaffolds, were found in literature and exhibited high mechanical properties, a promoted proliferation of cells, and even in some cases, shape-memory properties [16,17,30,32,33,34,35].

Nevertheless, in order to print scaffolds with adequate properties to be used in the biomedical sector, it must be taken into account that one of the disadvantages of conventional FFF is that it often requires raw materials in the form of filament spools. This leads to the use of usual commercial thermoplastic grades that cannot be tuned, few of which are suitable for use in biomedical applications. Mondal et al. modified the surface of their 3D printed PLA scaffolds with HA particles post printing [36]. However, to modify the matrix in its bulk and develop specific formulations, the production of filament spools by pellet extrusion is one of the solutions. Nevertheless, it requires specific equipment, adds a step to the manufacturing process, and often degrades polymers, especially in the case of hydrolysis-sensitive polymers. Formulations developed by solvent casting prior to filament extrusion have also often been seen, but this technique can only be considered on a laboratory scale and is not environmentally friendly [37,38]. Moreover, the materials used in biomedicine must be certified and cannot be modified in any way to make them printable, as modifications will revoke the certification [39]. Other processes derived from FFF directly allow the use of pellets, one of them being the ARBURG Plastic Freeforming process (APF), which allows to shape thermoplastic polymer or composite formulations specially adapted to a given use.

The APF process is an injection-based AM technique that consists in the deposition of individual droplets by a discharge unit equipped with a piezo actuator. It was already used in Hentschel et al.’s study, who qualified medical grade poly(methyl methacrylate) and obtained geometrically accurate specimens [39]. Precision in the production of parts is indeed a key element in the biomedical sector. Since APF is a process derived from injection, the flow rate and therefore the volume of the drop deposited is better controlled than with FFF (i.e., the volume of material deposited per unit of time is better controlled by pushing the material through a piston than by rotating an extrusion screw in a barrel).

The originality of this work thus lies in the way of producing scaffolds via the APF process. Indeed, a customized formulation of PLA and HA was prepared and used to print scaffolds for bone regeneration. First, the morphology of the scaffolds was investigated. Then, their biological properties were studied in terms of cell adhesion and proliferation. Finally, the biodegradability of the scaffolds was explored, as a function of their composition and time in cell culture medium at body temperature. To witness biodegradation, several features were followed with time, such as the molecular weight, the thermal properties, and the mechanical properties of the scaffolds after conditioning.

## 2. Materials and Methods

### 2.1. Materials

PLA Luminy LX575 pellets (98% L) were purchased from Total-Corbion PLA bv, the Netherlands. This grade has a glass transition temperature (T_g_) of 60 °C and a melting temperature (T_m_) of 165 °C. Hydroxyapatite (Ca_10_(PO_4_)_6_(OH)_2_) nanoXIM HAp203 particles with a size (d_50_) of 10 µm were supplied by Fluidinova, Portugal.

Pellets were dried in a vacuum oven at 80 °C overnight. Pure PLA and the composite formulation made of PLA and 10 wt.% of HA (PLA_90_/HA_10_) were then extruded by means of a Coperion twin-screw extruder (ZSK26MC) (ratio length (L) over diameter (D) L/D = 40). The screw speed was set at 120 rpm and the temperature profile was fixed between 180 and 210 °C from the feeder to the die. At the die, the extruded strand was cooled down in a water bath and pelletized.

After the extrusion step, the amount of HA in the pellets was checked by pyrolysis at 600 °C for 2 h and was reported in Table 1.

The density of the materials was measured using the buoyancy method, based on Archimedes’ principle (ISO 1183-1 Method A-Immersion method) on injection-molded samples. These samples were prepared with a Thermo Scientific HAAKE Minijet II injection molding press at 215 °C with an injection pressure of 200 bar during 6 s and a packing pressure of 30 bar for 6 s.

The injected samples made of pure PLA and PLA_90_/HA_10_ were weighed in water and in air with a METTLER TOLEDO laboratory scale and a density kit. Density was calculated following Equation (1):

(1) $$\rho_{s}=\frac{m_{s,a}\times \rho_{w}}{m_{s,a}-m_{s,w}}$$

with $\rho_{s}$ being the density of the sample, $m_{s,a}$ and $m_{s,w}$, the masses of the sample in air and in water, respectively, and $\rho_{w}$, the density of water (i.e., 1).

### 2.2. Scaffold Fabrication

**Figure 1:**
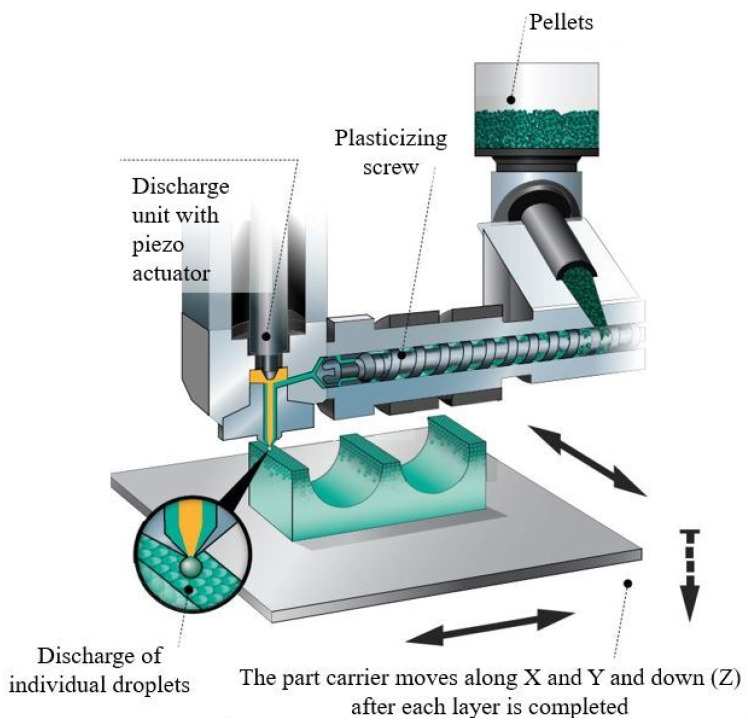
ARBURG Plastic Freeforming (APF) process (adapted from arburg.com).

A Freeformer machine with a 200 µm nozzle diameter was used to produce the scaffolds. Its specific operating system is shown in Figure 1.

The optimum printing conditions such as the nozzle temperature, the discharge parameter, the infill ratio, the slicing distance, the number of contours and the chamber temperature were investigated following the same qualitative approach as in previous work from Charlon and Soulestin [40]. Printability was evaluated in terms of pressure at the tip of the nozzle, aspect of the material during printing, and ease of removal of the part from its support after printing. The scaffolds were then visually inspected and their general porosity was assessed to define the reference parameters to be used to print scaffolds with satisfying characteristics. Finally, a nozzle temperature of 200 °C was chosen to print the scaffolds made of pure PLA. The PLA_90_/HA_10_ formulation was printed at 210 °C to avoid nozzle clogging, as the particles made the composite more viscous than pure PLA. A discharge parameter of 100% (i.e., the discharge parameter can be associated with the volume of material passing through the nozzle during each opening of the nozzle), an infill of 20%, a slicing distance of 165 µm as well as a chamber temperature of 30 °C were set with regard to the aspect of the printed scaffolds.

**Figure 2:**
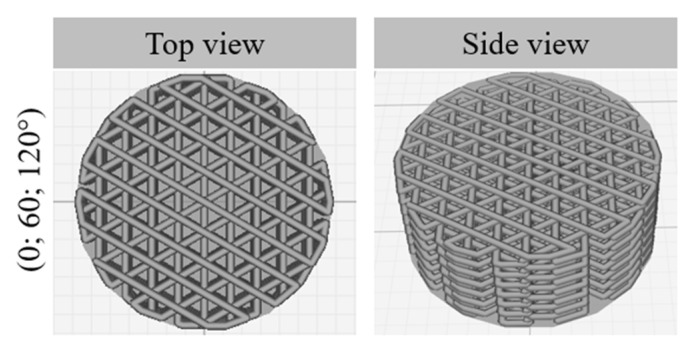
Top and side views of the scaffolds (lay-down patterns).

The pellets (PLA or PLA_90_/HA_10_) were fed into the hopper of the 3D printer and the molten polymer was then discharged as droplets strings [40] through the nozzle to form cylinder-shaped scaffolds. Their diameter was 14 mm for a height of 5 mm. The scaffolds were designed without contours (Figure 2) to help the culture medium enter the inner structure of the scaffolds and to allow good exchanges with the cells.

A lay-down pattern with orientations of (0; 60; 120°) was chosen, as it is often used for medical applications and this pattern was proven to result in the best properties among all the analyzed architectures in Ostrowska et al.’s study [41].

### 2.3. Scaffold Morphology and Surface Characteristics

Scaffold morphology was first assessed by optical microscopy (OM) as well as scanning electron microscopy (SEM) by means of a LEICA DM RXP and a JEOL JCM-6000 Plus versatile benchtop scanning electron microscope.

Scaffold porosity was determined by weighing the scaffolds and comparing their measured mass to the theoretical mass of full cylinders made from the same materials.

Their roughness was assessed by means of a Mahr MarSurf PS1 roughness-measuring instrument on 3 injection-molded samples of each formulation (PLA and PLA_90_/HA_10_), as this equipment was not adapted to direct measurements on scaffolds. A total of 3 measurements were done on each sample (9 measurements per formulation).

Water contact angle measurements were performed using a common sessile drop technique on these same 3 samples per formulation, with a KRUSS DSA 100S goniometer. 6 measurements were performed for each formulation.

### 2.4. Biological Studies

Scaffolds were washed with 70% ethanol for 15 min in 24-well plates. The plates were placed under a laminar flow hood to allow evaporation of the remaining ethanol. They were then decontaminated under a UV lamp for 20 min before the cell proliferation and degradation studies. In the following, decontaminated samples were referred to as PLA-d and PLA_90_/HA_10_-d in the text. PLA-d and PLA_90_/HA_10_-d scaffolds were placed in 24-well plates treated for low cell binding surface (Nunclon Sphera Surface, Thermo Scientific) for the cell proliferation study. Mesenchymal stromal cells from dental pulp (MSCs) of 8 independent donors were isolated [42] and amplified at a density of 3 × 10^3^ cell/cm^2^ in α-MEM (Lonza) supplemented with 10% heat-inactivated (56 °C, 30 min) fetal bovine serum (FBS), 1% penicillin/streptomycin/amphotericin B (PSA), and 1% glutamax (*v*/*v*, Gibco) and maintained in a humidified atmosphere of 5% CO_2_ at 37 °C with a medium change every two days. MSCs were seeded at a density of 2 × 10^4^ cells per scaffold and were cultured for 21 days. MSCs viability study and DNA extraction were performed at day 7, day 14, and day 21 on MSCs in accordance with the manufacturers’ protocols. For WST-1^®^ cell viability assay (Roche Diagnostics, Meylan, France), absorbance was measured at 440 nm using a FLUOstar Omega microplate reader (BMG Labtech, Ortenberg, Germany) against a background control as blank. A wavelength of 750 nm was used as a correction. DNA was extracted from the samples using MasterPure^TM^ DNA Purification Kit (Epicentre^®^ Biotechnologies, San Diego, CA, USA). The quantity of extracted DNA was then assessed by measuring the absorbance at 260 and 280 nm (Nanodrop^®^, Thermo Scientific, Waltham, MA, USA) with 260/280 nm absorbance ratio for all measured samples comprised between 1.8 and 2.

The morphology of MSCs was evaluated following cytoskeleton labeling after 21 days of culture on both scaffolds. Briefly, MSCs were seeded at a density of 2 × 10^4^ cells per scaffold. The scaffolds were then placed in 24-well plates treated for low cell binding surface. After 21 days of culture, cells were fixed with 4% (*w*/*v*) paraformaldehyde (Sigma-Aldrich, St. Louis, MO, USA) at 37 °C for 10 min and permeabilized with 0.5% (*v*/*v*) Triton X-100 for 5 min. Alexa Fluor-488-conjugated Phalloidin^®^ (1/100 dilution in 0.5% bovine serum albumin) was used to stain F-actin for 45 min at room temperature. Nuclei were counterstained with 4,6-diamidino-2-phenylindole (DAPI, 100 ng/mL, 1/3000 dilution) for 5 min. Stained MSCs were imaged by confocal laser scanning microscopy (CLSM, Zeiss LSM 710 NLO, ×20 objective, Numerical Aperture 1.4, Jena, Germany), and confocal reflectance mode was used on the same sample location to image scaffolds. 

### 2.5. Biodegradation Study

#### 2.5.1. Conditioning

To evaluate the biodegradability of the scaffolds in the body environment (ISO 13781-Biodegradability norm), scaffolds were placed in 10 mL of DMEM-Glutamax^®^ (Gibco) culture medium supplemented with 10% heat-inactivated FBS and 1% PSA. Scaffolds were maintained in a humidified atmosphere of 5% CO_2_ at 37 °C with a medium change every week. At different times (1, 3, and 5 months), scaffolds were removed from the medium and kept in a freezer (−20 °C). The extensions −1 m, −3 m, and −5 m were added after the sample names and correspond to the duration of conditioning in the medium, namely 1 month, 3 months, and 5 months, respectively. At the end of the degradation study, all the samples were characterized to evaluate their properties.

#### 2.5.2. Gel Permeation Chromatography

Gel permeation chromatography (GPC) analyses were performed in CHCl_3_ at 30 °C using an Agilent liquid chromatograph equipped with an Agilent degasser, an isocratic HPLC pump (flow rate = 1 mL/min), an Agilent autosampler (loop volume = 100 µL, solution conc. = 1 mg/mL), an Agilent DRI refractive index detector, and three columns: a PL gel 10 µm guard column and two PL gel Mixed-D 10 µm columns (linear columns for separation of MWPS ranging from 500 to 10^7^ g/mol). Polystyrene standards were used for calibration. GPC analyses were performed for the different formulations before/after extrusion and after scaffold production, decontamination, and conditioning. Three samples were tested for each condition.

#### 2.5.3. Thermal Analysis

Differential scanning calorimetry (DSC) scans were performed on similar samples as for the GPC study. The DSC analyses were carried out with a DSC Q2000 from TA Instruments^®^, New Castle, DE, USA. Enthalpy and temperature calibrations were performed using an indium standard. Around 5–10 mg of samples were sealed in T_zero_ pan and heated from −80 °C to 200 °C at 10 °C/min under nitrogen atmosphere. The characteristic temperatures such as the glass transition temperature (T_g_), cold crystallization temperature (T_cc_), and melting temperature (T_m_) were collected to observe the variations of these features with the amount of HA and the processing steps. 

The degree of crystallinity χ(%) was determined following Equation (2):

(2) $$\chi \left(\%\right)=\frac{\Delta H_{m}-\Delta H_{cc}}{\left(1-wt{\%}_{HA}\right)\times \Delta H_{m}^{{}^{\circ}}}$$

where $\Delta H_{m}$ is the melting enthalpy, $\Delta H_{cc}$ is the enthalpy of cold crystallization, $wt{\%}_{HA}$ is the weight fraction of HA particles, and $\Delta H_{m}^{{}^{\circ}}$, the melting enthalpy of the 100% crystalline polymer (i.e., 93.7 J/g for PLA) [43]. Three samples were tested for each condition.

#### 2.5.4. Mechanical Properties

The compression properties of the scaffolds were studied by means of a Zwick/Roell Z010 following the standard method NF EN ISO 604 for compressive properties of plastics. Samples were maintained at 37 °C in the oven of the machine for 5 min before testing at a speed of 1 mm/min at this same temperature. The compression modulus and compressive strength at yield (or flexure point) were determined from the stress–strain curves. At least five specimens were tested for each condition. The properties of the scaffolds after the degradation study were also investigated at each point in time (1, 3, and 5 months) to investigate the probable decrease of the mechanical properties of the scaffolds with degradation.

### 2.6. Statistical Analysis

All results were represented as histograms (mean ± standard deviation (SD)). Statistical analyses were performed using GraphPad Prism^®^ software (version number 5, San Diego, CA, USA): a non-parametric Mann–Whitney test for independent samples was applied for biological studies, determination of scaffold swelling, and mechanical properties before and after conditioning; an unpaired *t*-test was applied for GPC analyses. For each test, a value of *p* < 0.05 was accepted as statistically significant *p* (rejection level of the null hypothesis of equal means).

## 3. Results and Discussions

### 3.1. Scaffold Morphology and Surface Characteristics

To ensure proper cell seeding and fluid exchange inside and outside the scaffolds, certain features are required, such as a high porosity of the entire scaffold and interconnectivity of the pores. In order to achieve such characteristics, composite scaffolds made of PLA with 10 wt.% of HA (i.e., PLA_90_/HA_10_) were produced with the APF process and PLA scaffolds (i.e., PLA) were produced as a reference. 

**Figure 3:**
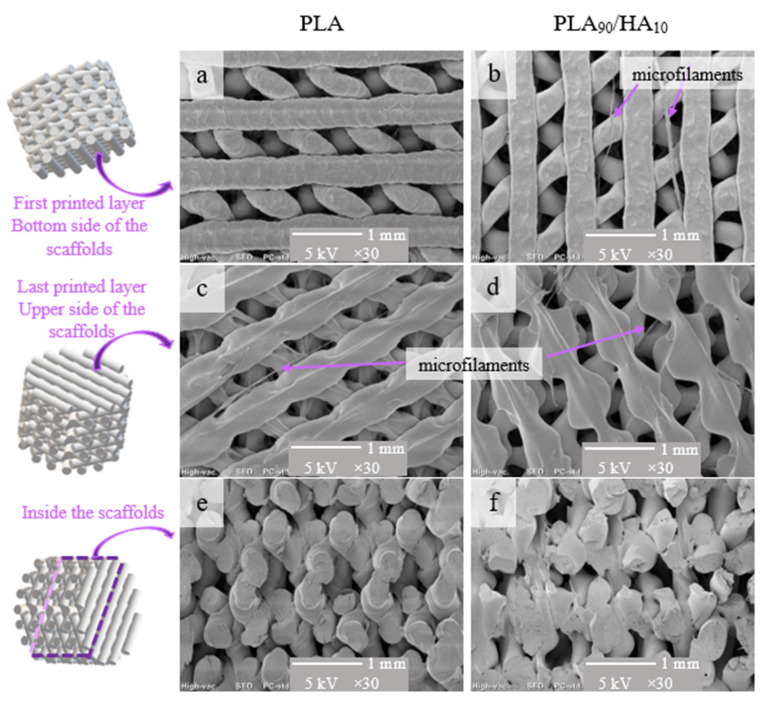
Schematic representation of the areas of the scaffolds (left) and SEM pictures (right) of the different zones of the scaffolds; (**a**,**c**,**e**): PLA scaffolds and (**b**,**d**,**f**): PLA_90_/HA_10_ scaffolds. Their structure is the same in terms of lay-down pattern but depending on the orientation of the samples in the SEM sample holder, the orientation of the filament in the pictures may appear different.

The results of SEM observations of the two 3D-printed PLA and PLA_90_/HA_10_ scaffolds (lay-down pattern with orientations of (0; 60; 120°)) are shown in Figure 3.

**Figure 4:**
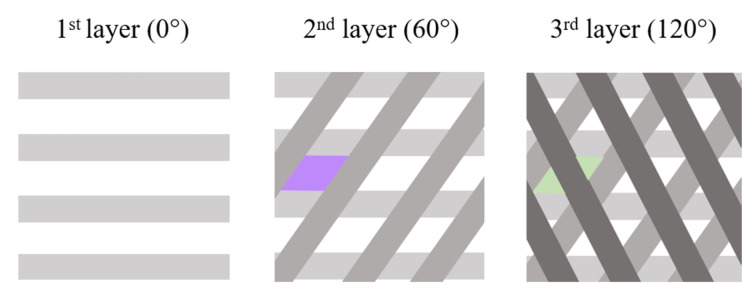
Schematic representation of the layer deposition until layer 3. A diamond shape is highlighted in purple in the 2nd layer, and the resulting triangles are highlighted in green in the 3rd one.

Incrementing the deposition angle by 60° with each layer resulted in complex shaped porosities. Between the first 2 deposited layers (Figure 3a,b), they almost look like diamonds with sides measuring about 500 µm, while between the first 3 deposited layers, they look like triangles with sides measuring between 100–300 µm (Figure 4). 

Due to the successive increment of the deposition angle, one could imagine that the channels are closed after 4 layers. However, in the 3D scaffolds, these channels are open and interconnected (i.e., the first 3 deposited layers are not in the same plane in the 3D scaffold (Figure 3e,f)). In fact, because the beads are not in the same plane, this creates a high interconnectivity of the pores and generates a tortuous network. This is a very important feature to ensure good oxygen and nutrient diffusion into the scaffold [44]. Knowing that the largest pore sizes measured (500 µm) concern the diamond-shaped pores created between the first 2 layers and that the smallest pores measured (200–300 µm) correspond to the triangular pores created between the first 3 layers, sizes between 100 and 600 µm are well obtained, as advised by Abbasi et al. to reach a good regeneration and mineralization [7]. 

Although the measured density of the PLA_90_/HA_10_ material (1.314) is higher than that of pure PLA (1.251), the density of the PLA_90_/HA_10_ scaffolds (0.891) was found to be lower than that of PLA scaffolds (0.935), demonstrating a higher porosity of the PLA_90_/HA_10_ scaffolds. General porosities of 25% and 32% were assessed for pure PLA scaffolds and PLA_90_/HA_10_ scaffolds, respectively. As seen in the SEM pictures (Figure 3c,d), the material appears more spread in the last layers of PLA_90_/HA_10_ scaffolds, which may mean that PLA_90_/HA_10_ got more fluid during printing than pure PLA. HA may have induced degradation in the material because of its hydrophilic nature, generating hydrolysis (polymer chain scission) and thus, a decrease in the viscosity. In the APF process, a high material fluidity leads to a lower pressure applied on the molten material to pass through the nozzle and, thus, to less material deposition. In addition, the increasing hydrolysis of the material during printing induced variations in the discharge parameter (i.e., the material flow passing through the nozzle), which tended to oscillate and sometimes decrease up to 90%, again leading to less material deposition. 

**Figure 5:**
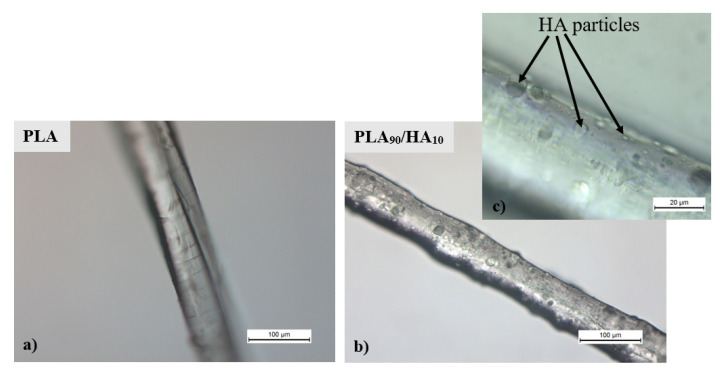
OM pictures of microfilaments of (**a**) a pure PLA filament and (**b**) a PLA_90_/HA_10_ filament, and (**c**) close up on a PLA_90_/HA_10_ filament.

Microfilaments resulting from printing burrs were observed in the SEM pictures (Figure 3). These microfilaments could promote the even more tortuous aspect of the scaffolds, preventing cells from being dragged out of the scaffolds by exchanges with the environment or, on the contrary, delaying the colonization of the scaffolds due to the difficulties to penetrate them. OM observations on such microfilaments can be seen in Figure 5. Logically, HA particles are present in the PLA_90_/HA_10_ microfilament, whereas the PLA microfilament is smooth. The particles seem to make the PLA_90_/HA_10_ filament rougher (Figure 5) and the average particle size announced by the manufacturer (10 µm) seems legitimate.

In addition, Figure 5c shows that HA particles are well-dispersed, and no aggregates were noticed on the samples. The roughness measurement revealed an increase in the roughness (Rz) of the PLA_90_/HA_10_ samples of 26% (1.29 µm for pure PLA and 2.92 µm for PLA_90_/HA_10_), which seems in accordance with the OM pictures.

**Figure 6:**
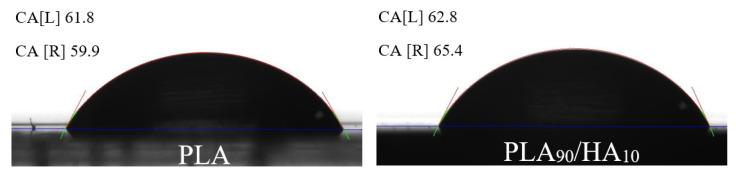
Water contact angle of PLA (**left**) and PLA_90_/HA_10_ (**right**).

The results of water contact angle (CA) did not show any significant differences between the two materials (Figure 6).

The water CA of pure PLA was found to be of 60.45 ± 2.19, whereas that of PLA_90_/HA_10_ was found to be slightly higher with a value of 62.57 ± 4.04. This is surprising because HA is supposed to increase hydrophilicity [45] but the increased roughness may prevent the drop from spreading. In addition, the HA particles are probably partially coated with PLA, so logically this gives similar values. The behavior of the materials will likely change with degradation.

Although the morphology of the scaffolds seems to be suitable for cell colonization, their biological properties need to be studied to consider them as implants.

### 3.2. Biological Properties

**Figure 7:**
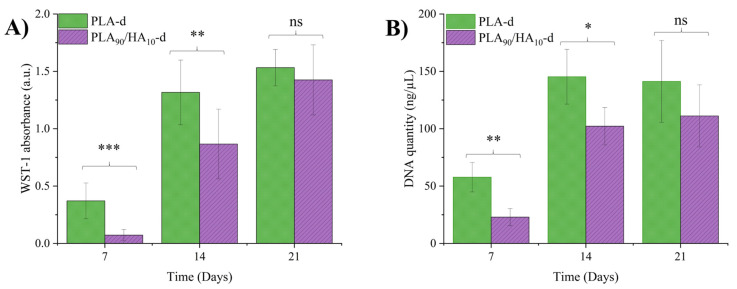
WST-1 absorbance (**A**) and DNA quantification (**B**) of MSCs on day 7, day 14, and day 21 of culture in PLA-d and PLA_90_/HA_10_-d scaffolds (mean ± SD). Mann–Whitney test, * *p* < 0.05, ** *p* < 0.005, *** *p* < 0.0005, ns = non-significant.

While PLA and HA are both known to be biocompatible, developing an appropriate scaffold with good cell adhesion and proliferation properties is one of the key elements in regenerative medicine [10,11,12,13]. Mesenchymal stem cells (MSCs) from dental pulp were therefore cultured on decontaminated (-d) PLA and PLA_90_/HA_10_ scaffolds (i.e., PLA-d and PLA_90_/HA_10_-d) in plates treated for low cell binding surface, in order to avoid cell adhesion elsewhere than on the scaffolds. The cell proliferation within the scaffolds was monitored by WST-1 and DNA quantification on day 7, 14, and 21 of culture, using independent samples for each test and time point (Figure 7). While WST-1 absorbance reflects cell viability, DNA quantification is correlated to the number of MSCs. 

Surprisingly, both WST-1 absorbance and DNA quantity of MSCs on day 7 and day 14 showed significantly higher values for PLA-d in comparison with PLA_90_/HA_10_-d. This reflects a higher number of MSCs within PLA-d scaffolds during the first 14 days of culture. This result suggests a better initial adhesion of MSCs on PLA-d scaffolds, whereas both materials exhibited the same hydrophilicity, which is crucial for initial cell adhesion [46]. However, although PLA_90_/HA_10_ was hydrophilic and the particles enhanced the roughness of the material, the difference in initial adhesion could be attributed to the fluidity of PLA_90_/HA_10_ during processing. This led to variations in material deposition in some areas, and thus, scaffolds with altered features (microfilaments, deformed pores, irregularities, lack of/too much material), which may have delayed cell adhesion. Moreover, HA particles were probably coated with PLA for the first 14 days, which may have reduced the effect of HA at the beginning of the test.

After 21 days of culture, no significant differences were observed in WST-1 absorbance and DNA quantity values between both scaffolds. This result might reflect the degradation of PLA_90_/HA_10_-d scaffolds, leading to the accessibility of HA particles to the cells. Moreover, an increase in cell viability and DNA quantity over time was observed for both scaffolds. Taken together, these results suggest a good proliferation of MSCs within both biocompatible scaffolds, in spite of a better initial cell adhesion within PLA-d scaffolds.

**Figure 8:**
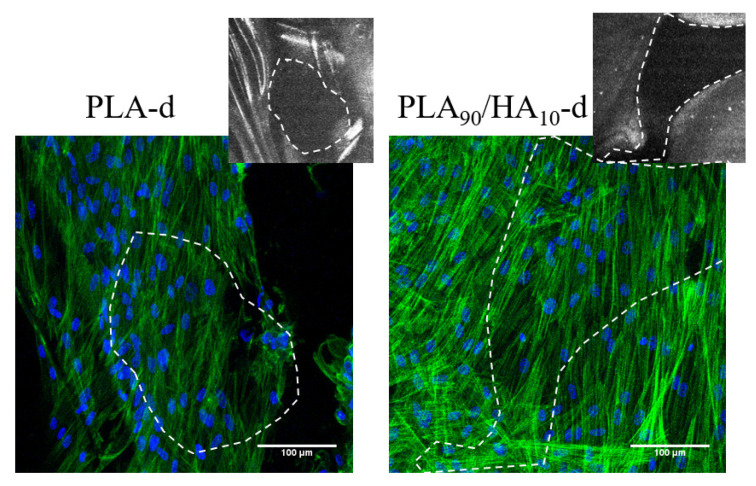
Confocal microscopy visualization of MSCs cultured on PLA-d and PLA_90_/HA_10_-d for 21 days, labeled with Phalloidin^®^ (green; cytoskeleton) and DAPI (blue; nucleus). Scale bars = 100 µm; insert = Scaffolds visualization in reflexion mode. The white dotted delimitations highlight the corresponding scaffold pores.

After 21 days of culture on both scaffolds, MSCs were fixed and labeled with Phalloidin^®^ and DAPI, which allowed the identification of MSCs morphology, by observing their cytoskeleton and nucleus, respectively. Observation of labeled MSCs on the scaffolds under a laser scanning confocal microscope confirmed the presence of MSCs adhering to the scaffolds after 21 days of culture (Figure 8). Moreover, MSCs colonized the filaments of both scaffolds and created bridges through the pores of both scaffolds.

The scaffolds must degrade in the patient’s body during bone regeneration to avoid a second surgery. Therefore, the biodegradability of the scaffolds must be studied.

### 3.3. Biodegradability Study

**Figure 9:**
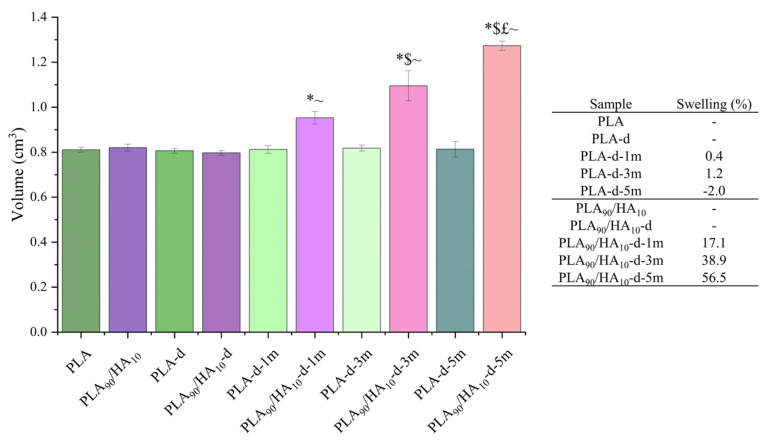
Volume (**left**) and related swelling (**right**) of the samples before/after conditioning in cell culture medium at 37 °C during up to 5 months. Mann–Whitney test *p* < 0.05; for PLA or PLA_90_/HA_10_: * vs. -d, $ vs. 1 m, £ vs. 3 m; for PLA_90_/HA_10_: ~ vs. PLA.

After conditioning in cell culture medium at 37 °C during up to 5 months, according to the ISO 13781-Biodegradability norm, the dimensions of the scaffolds were measured. Their volume was then determined and compared to their initial volume (i.e., before conditioning = 0 months). The results of these measurements can be seen in Figure 9.

After 5 months of conditioning, an increase in the volume of PLA_90_/HA_10_ scaffolds of more than 50% was observed, while the pure PLA scaffolds kept an unchanged volume. This is explained by the fact that HA is very hydrophilic and attracted the water contained in the cell culture medium into the network of PLA_90_/HA_10_ scaffolds. Indeed, the addition of HA particles increased swelling with conditioning time. This difference in water penetration certainly had an impact on the degradation of the scaffolds, and this will be verified by the following tests.

**Figure 10:**
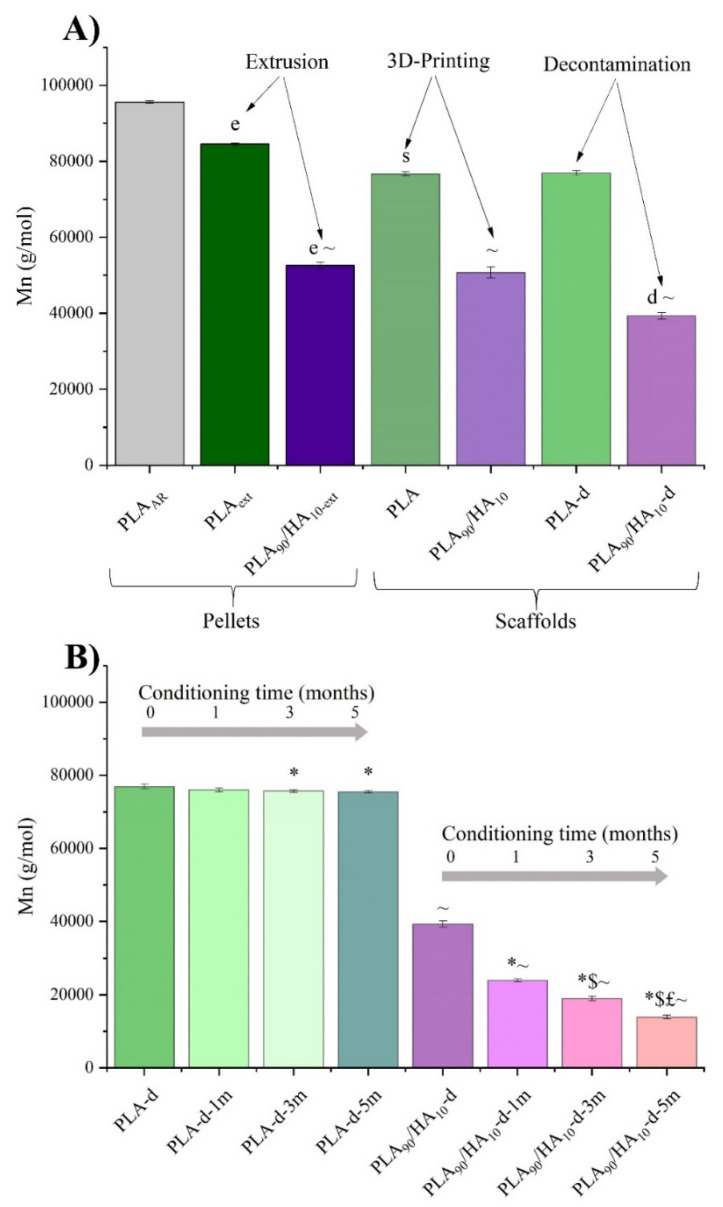
Average molecular weight in number (**A**) from the as-received pellets to the decontaminated scaffolds; (**B**) of the scaffolds during conditioning. Unpaired *t*-test *p* < 0.05; significant effect of: extrusion (e), 3D printing (s), decontamination (d); for PLA or PLA_90_/HA_10_: * vs. -d, $ vs. -1 m, £ vs. -3 m; for PLA_90_/HA_10_: ~ vs. PLA (of the same condition).

During processing, UV-decontamination and then conditioning for the biodegradation study, chain scission is likely to happen to PLA, as a result of thermo-mechanical stress, radiation, and hydrolysis. GPC is an analytical technique that enables the measurement of the molecular weight of polymer samples and, therefore, allows the evaluation of polymer degradation. GPC was performed on the as-received pellets (PLA_AR_), the extruded formulations (PLA_ext_ and PLA_90_/HA_10-ext_), the two types of scaffolds after 3D-printing (PLA and PLA_90_/HA_10_), after decontamination (index -d), and after conditioning in cell culture medium up to 5 months (indexes −1 m, −3 m and −5 m). These GPC results can be seen in Figure 10.

PLA_AR_ pellets had an average molecular weight in number (Mn) of 95,571 g/mol and their extrusion significantly affected their Mn. It decreased from 95,571 g/mol for PLA_AR_ to 84,493 g/mol and 52,570 g/mol for PLA_ext_ and PLA_90_/HA_10-ext_, respectively. These results are in accordance with Backes et al.’s study who showed that melt processing had a great impact on the Mn of PLA and that the presence of calcium (such as in HA) induced more thermo-degradation [47]. Indeed, since HA is very hydrophilic, the material was able to re-absorb moisture faster than pure PLA and the high temperature processing may have induced hydrolysis reactions and, thus, polymeric chain scission. Then, the transformation of the extruded pellets into 3D-printed scaffolds also generated little degradation because the ARBURG Plastic Freeforming (APF) process induces additional thermo-mechanical stresses to the materials.

Before biological testing, the scaffolds were exposed to UV, but this step did not cause great degradation to the bulk of both scaffolds. Then, conditioning clearly affected the scaffolds containing HA. Their Mn decreased gradually with the conditioning duration in culture medium at 37 °C to reach a value of 13,930 g/mol after 5 months (i.e., decrease of 65% compared to PLA_90_/HA_10_-d). PLA scaffolds, however, showed very little degradation with conditioning (i.e., Mn of 75,499 g/mol after 5 months, equivalent to a decrease <2% compared to PLA-d), confirming that HA is of great help to improve biodegradability of PLA.

**Figure 11:**
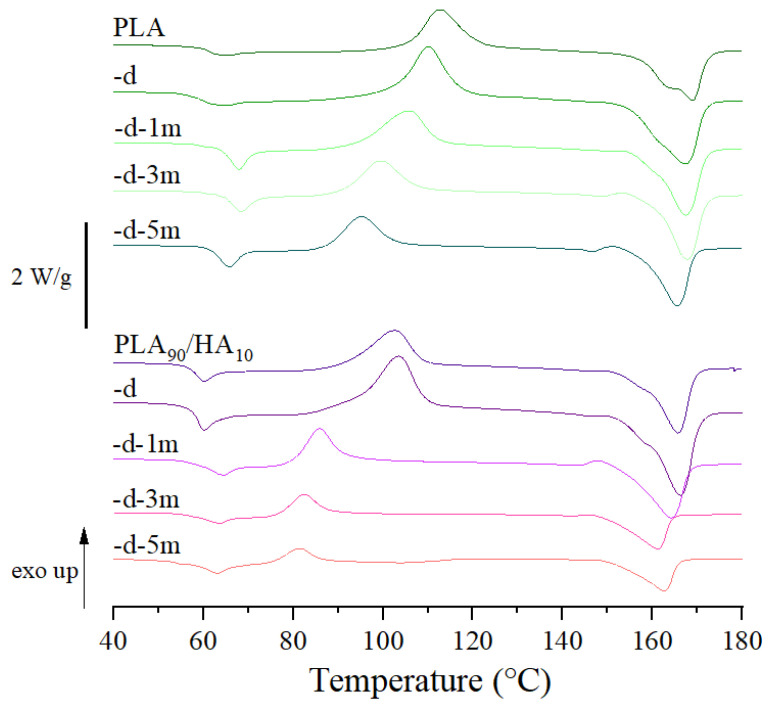
Representative DSC thermograms of the scaffolds after processing, decontamination, and conditioning in cell culture medium at 37 °C up to 5 months.

DSC was carried out on the scaffolds (Table 2 and Figure 11) to provide additional information to the GPC results and in particular by noting the influence of the chain size on the thermal properties of the different materials and scaffolds. Only the first heating step was commented since it represents the actual state of the materials, and thus of the scaffolds (i.e., the second heating step erases the thermal history).

The crystallinity degree of the scaffolds after AM was low in both cases, since they were rapidly cooled down from the nozzle temperature to the manufacturing chamber temperature (30 °C) to avoid crystallization, which is known to impair biodegradation [48,49].

Concerning PLA scaffolds, it can be seen that their T_g_ slightly increased with conditioning, confirming that conditioning did not induce any decrease in the length of PLA chains. An endothermic peak can be noticed at the T_g_ and is mostly visible in the case of conditioned samples. It corresponds to an enthalpy relaxation peak. Indeed, PLA is sensitive to physical aging at temperatures below but close to T_g_ and conditioning at 37 °C must have induced structural relaxation towards equilibrium [50]. Physical aging is also assumed to induce a decrease in the free volume and to increase T_g_ [51], justifying the evolution of T_g_ towards higher temperatures in the first heating step. The cold crystallization temperature (T_cc_) however decreased from 111.9 °C to 95.3 °C. This may be because of the organization of the polymer chains into nuclei during conditioning, favoring germination upon heating during DSC testing. Such behavior was already seen in literature, such as in Na et al.’s study, where morphological observations revealed a high density of nuclei in PLLA annealed below T_g_ (50 °C) [52]. In the present study, conditioning at 37 °C can be considered as annealing below T_g_. 

In contrast, for PLA_90_/HA_10_ scaffolds, not only physical aging occurred but also biodegradation that was clearly catalyzed by the presence of HA. Indeed, T_g_ progressively decreased with the duration of conditioning (from 56.3 °C to 50.3 °C), which is a sign of greater chain mobility. This may have been induced by a reduced Mn and the plasticizing effect of water [53,54], which penetrated the scaffolds more easily due to HA hydrophilicity. Their T_cc_ was already lower (103.2 °C) than that of PLA scaffolds, even without any conditioning in the culture medium. This may be because HA induced heterogeneous nucleation into the PLA matrix [55], but also because a greater degradation of the scaffolds occurred during processing (i.e., lower Mn increase chain mobility and favor crystallization at lower temperatures), which is in line with the GPC results. T_cc_ decreased with conditioning until it reached 78.8 °C for PLA_90_/HA_10_-d-5 m and the degree of crystallinity increased, because degradation primarily affects the amorphous regions and the polymer chains have reorganized into nuclei, as for PLA scaffolds. The wide and spread melting peaks are another indication of degradation that led to smaller (decreased melting temperature (T_m_) onset) and more heterogeneous populations of crystals in terms of size.

**Figure 12:**
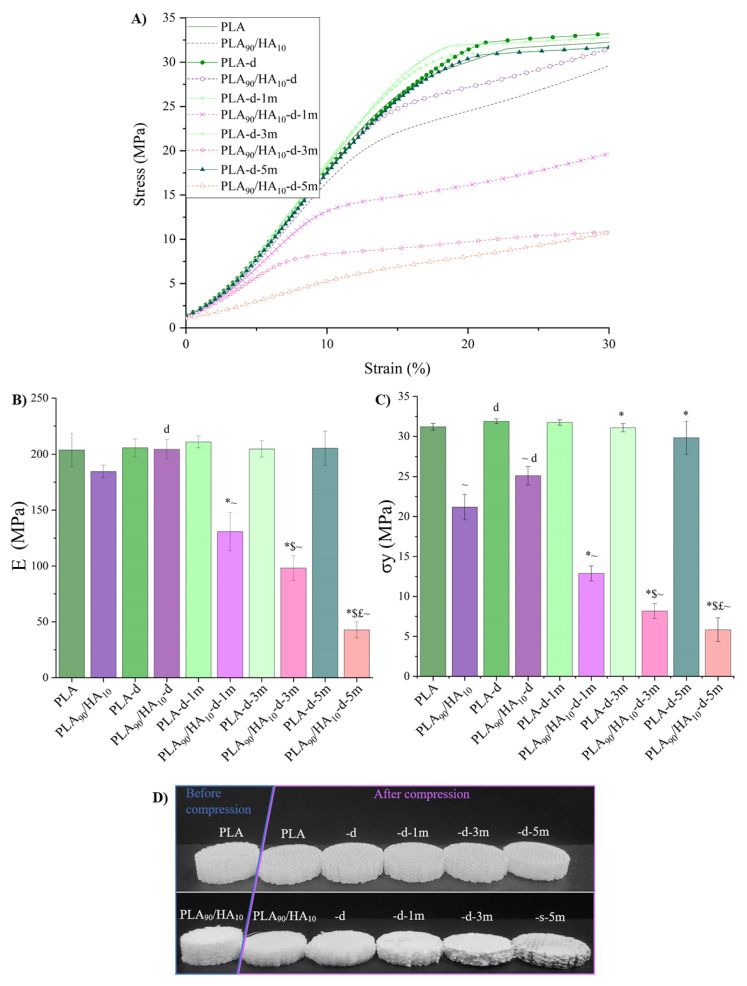
Mechanical properties in compression at yield: (**A**) Compression curves; (**B**) compression modulus; (**C**) compression strength at yield (or flexure point); (**D**) aspect of the scaffolds before/after compression. Mann–Whitney test *p* < 0.05; significant effect of decontamination (d); for PLA or PLA_90_/HA_10_: * vs. -d, $ vs. -1 m, £ vs. -3 m; for PLA_90_/HA_10_: ~ vs. PLA (of the same condition).

Mechanical tests in compression (Figure 12) were performed on the scaffolds after all the steps mentioned above in order to correlate the observed results with the behavior of the scaffolds at the macroscopic scale. 

For the non-degraded PLA and PLA_90_/HA_10_, the elastic moduli are comparable (204 MPa for PLA and 185 MPa for PLA_90_/HA_10_), but the compressive strength of PLA_90_/HA_10_ decreased with the addition of HA (21.2 MPa for PLA_90_/HA_10_ against 31.2 MPa for PLA). This confirms the decrease in Mn seen in GPC and thus, a premature yield because of fewer entanglements between the polymer chains. Values found in the literature range between 1.5 MPa and 45 MPa for the compressive strength of cancellous bone and 20 MPa and 50 MPa for that of apatite cements, suggesting that both scaffolds would be suitable for bone regeneration applications [56]. 

After conditioning, the difference with PLA_90_/HA_10_ scaffolds is that the presence of HA (hydrophilic) decreased the Mn of the PLA matrix and the number of entanglements between the chains and made the scaffolds swell (Figure 9). This must have created many defects and gaps in the scaffolds, decreasing the cohesion between the printed filaments. Their compression modulus and strength became significantly lower than those of PLA scaffolds, because their macroscopic network did not resist the applied force but collapsed completely. Indeed, it is known that PLA tends to become increasingly brittle as its Mn decreases below 40,000 g/mol [57]. Here, the Mn of PLA_90_/HA_10_ scaffolds was already at the limit after decontamination (39,421 g/mol) and decreased gradually with conditioning time. At the end of the test, the degraded PLA_90_/HA_10_ scaffolds, which were compressed until reaching a height of 2 mm, remained this way when stress was removed as can be seen in Figure 12D (i.e., plastic deformation and fragile fracture occurred). On the contrary, the PLA samples almost recovered their initial dimensions (elastic buckling). 

These results again confirm that biodegradation occurred for PLA_90_/HA_10_ scaffolds, while PLA scaffolds did not significantly biodegrade under these conditions, as they retained their mechanical properties even after 5 months of conditioning.

The fact that PLA scaffolds did not biodegrade at all is a little appealing because PLA is supposed to be fully biodegradable in the body. The major reason for this incomplete biodegradation is that the medium was changed regularly, removing the liberated lactic acid generated by PLA degradation. In the case of PLA_90_/HA_10_ scaffolds, degradation must have been increased so much thanks to the hydrophilicity of HA particles that degradation still being encouraged. Degradation would probably have been even more important without medium removal. Normally, lactic acid induces an autocatalytic effect of hydrolysis degradation [58]. 

## 4. Conclusions

In this study, two types of materials were chosen to produce scaffolds for bone regeneration. PLA and a formulation of PLA containing 10 wt.% of HA (PLA_90_/HA_10_) were first extruded and pelletized to be used as feeding materials to 3D-print scaffolds via a fused filament fabrication (FFF)-derived process (APF process). The two types of scaffolds were decontaminated, and their biological properties were assessed. Scaffolds were then conditioned in cell culture medium at 37 °C during up to 5 months to mimic the body environment and to study their biodegradation.

The results show that:

- All the scaffolds were biocompatible (not cytotoxic) and led to cell proliferation.
- Cell adhesion at the beginning of the test was better on pure PLA scaffolds, which was surprising since HA was supposed to increase cell adhesion because of increased roughness and hydrophilicity. Nevertheless, after 21 days of culture, both scaffolds were colonized by a similar number of cells, meaning that proliferation in PLA_90_/HA_10_-d occurred efficiently.
- GPC, DSC, and the results of compression tests revealed that the presence of HA greatly affected the molar mass (Mn) of the PLA matrix in the PLA_90_/HA_10_ samples, even before the degradation study. This can be explained by HA hydrophilicity that induced moisture absorption during processing, and thus, hydrolysis.
- The mechanical properties of the PLA_90_/HA_10_ scaffolds were still found to be suitable for bone regeneration applications, because of their adequate compressive properties that remained high enough.
- The Mn of PLA_90_/HA_10_ scaffolds greatly decreased during conditioning, which is a good sign that this type of material and structure would biodegrade after implantation in the body, whereas pure PLA did not. This demonstrates the importance of adding HA to PLA to reach gradual degradation simultaneously with bone regrowth in vivo.
- Although HA did not improve cell attachment at first, probably because the particles were partially coated with PLA, it did improve their proliferation over time, suggesting that this superficial PLA coating was easily removed with degradation.
- The APF process permitted to obtain scaffolds with satisfying features from specially formulated pellets without the need to prepare 3D printing filament, avoiding further degradation of the formulation containing HA.

These encouraging results suggest the potential use of the developed scaffolds in applications such as alveolar bone reconstruction after periodontitis, where the scaffolds are expected to biodegrade simultaneously with cell proliferation. In addition, future research should focus on the fatigue behavior of the scaffolds to verify that their mechanical properties are maintained over multiple cycles, so that these scaffolds can be used in applications where the mechanical stresses are greater than in dentistry.

This paper also paves the way for future research, where it would be interesting to study the effect of HA on cell differentiation. Indeed, the presence of HA in a polymeric matrix mimics the natural environment of cells in bone and should help the cells to differentiate into osteoblasts.

## Figures and Tables

**Table 1:** Nomenclature of the samples, theoretical and experimental amounts of HA, and sample density.

Name of the Formulation	Amount of PLA (wt.%)	Theoretical Amount of HA (wt.%)	Experimental Amount of HA (wt.%)	Density
PLA	100	0	0	1.251
PLA_90_/HA_10_	90	10	9.55	1.314

**Table 2:** Onset glass transition temperature (Tg(onset)), cold crystallization enthalpy (ΔHcc), cold crystallization temperature (Tcc), melting enthalpy (ΔHm), melting temperature (Tm), and degree of crystallinity (χ ) of pellets and scaffolds as a function of their history and conditioning time in cell culture medium (up to 5 months).

Sample	Time (Months)	Tg (Onset) (°C)	ΔHcc (J/g)	Tcc (°C)	ΔHm (J/g)	Tm (°C)	χ (%)
PLA	0	56.9	35.8	111.9	40.3	168.7	5
PLA-d	0	57.3	31.3	111.2	38.1	167.9	7
PLA-d-1 m	1	57.0	33.9	106.0	39.0	168.2	5
PLA-d-3 m	3	57.4	35.8	99.6	42.0	167.7	7
PLA-d-5 m	5	58.6	36.4	95.3	45.2	165.9	9
PLA_90_/HA_10_	0	56.3	34.2	103.2	37.8	166.0	4
PLA_90_/HA_10_-d	0	56.9	32.8	103.1	39.0	165.8	7
PLA_90_/HA_10_-d-1 m	1	54.6	24.3	85.8	34.3	164.2	12
PLA_90_/HA_10_-d-3 m	3	54.0	19.9	82.7	29.6	161.6	12
PLA_90_/HA_10_-d-5 m	5	50.3	10.5	78.8	24.1	162.4	16

## Data Availability

The datasets generated during and/or analysed during the current study are available from the corresponding author on reasonable request.
